# Promoting Positive Social Interactions: Recommendation for a Post-Pandemic School-Based Intervention for Social Anxiety

**DOI:** 10.3390/children10030491

**Published:** 2023-03-02

**Authors:** Yang Ni, Fanli Jia

**Affiliations:** 1 School of International and Public Affairs, Columbia University, New York, NY 10027, USA; 2 Department of Psychology, Seton Hall University, South Orange, NJ 07079, USA

**Keywords:** intervention, social anxiety, post-COVID-19, school, social interaction

## Abstract

The purpose of this perspective article is to identify problematic behaviors during the COVID-19 pandemic and recommend a school-based intervention (e.g., self-reflection, motivational interview, and workbook) to address post-COVID social anxiety among children and adolescents. The recommendations involve comparing students’ social interaction behaviors pre-pandemic, during the pandemic, and post-pandemic, and evaluating any behavioral changes in social relationships six months later. We also discuss the evaluation criteria and surveys used to assess the impact of the intervention on behavioral changes. Our evaluation criteria are based on students’ beliefs and abilities and aim to demonstrate that the intervention improves in-person social interactions and helps students adapt to the transition back to school. The proposed perspectives and strategies of the intervention can be modified to meet the needs of the researchers and professionals. By working together, global policymakers from the fields of education and public health can create school-based interventions that enhance students’ physical, mental, and spiritual well-being. This program aims to mitigate the negative effects of school closures and social isolation and to broaden the role of schools in supporting students in the challenging post-pandemic world by addressing their holistic needs.

## 1. Introduction

Public health agencies and academic institutions globally are prioritizing the recovery of mental health in response to the major source of stress caused by the COVID-19 pandemic, which has affected the economic, political, social, and environmental aspects of our lives at both the individual and societal levels [1]. The purpose of this article is to examine a possible intervention option to address the negative effects of COVID-19 and improve the mental health and social relationships of young people, particularly in light of the pandemic’s longstanding effects on their mental and physical health. Specifically, this perspective article intends to address the issue of social anxiety caused by school closures and related social isolation among school-aged children.

Recent researchers reported that many social factors are still affecting young people’s mental health and well-being, although the significant global pandemic has approached its final stage from the public health measurement perspective [2]. Kathirvel [3] of the World Bank predicts that the COVID-19 pandemic will cause a decades-long global economic recession that may largely affect mental health, including correlations with distress, anxiety, depression, and substance abuse disorders. The Centers for Disease Control and Prevention (CDC) has reported that at least one adverse mental or behavioral health condition has been developed by 40.9% of US adult respondents, with 10.7% of respondents seriously considering suicide in the last 30 days [4]. Other researchers have also found a similar trend globally that children and adolescents are suffering from more serious depression and anxiety effects, with a 30–50% report rate during the COVID-19 pandemic [5].

Social isolation is a core factor that harms young people’s well-being, according to Hossain [6], among other influencing factors such as economic and physical health. The World Health Organization [7,8] suggests that COVID-19 can lead to significant mental health and psycho-social consequences, and that new pandemic response measures, including self-isolation and quarantine, have affected people’s usual activities and livelihoods and increased the possibility of mental illness. Research indicates that social isolation seriously affects most aspects of emotional health and causes significant deterioration in social relationships [9]. Moreover, people are avoiding social relations more often because of the fear of being infected by others [9,10].

The COVID-19 pandemic significantly impacts social relationships, not only through forced social isolation but also through social anxiety in its aftermath. The prolonged period of remote work and study makes many individuals nervous about returning to face-to-face interactions. Flaskerud [11] predicts that various psycho-social factors continue to contribute to increased anxiety and depression levels, with growing fear of social settings leading to increased stress levels. Avoiding social interactions can exacerbate anxiety over the long term. A psychiatry expert suggests that the COVID-19 pandemic may leave a legacy in the rising incidence of social anxiety disorder as well as loneliness and depression [12,13].

Many young people lack social support when it is most needed, and afterward, they experience anxiety that prompts them to avoid social interactions [14]. Therefore, this perspective article first focuses on identifying potential emotional and behavioral issues caused by the COVID-19 pandemic. Since the COVID-19 pandemic has caused long-standing effects on students’ mental health and harmed students’ social skills in establishing and maintaining young people’s social relationships [15], we recommend a school-based intervention to cope with post-COVID social anxiety by identifying problematic behaviors in social interactions. We recommend comparing young people’s social interaction behaviors before, during, and after COVID-19 to identify the problematic behavior of avoiding social interactions by measuring time spent on social media and in-person interactions. A comprehensive workshop-based intervention in schools can enhance in-person social interactions and foster positive relationships among students, applying the participation principle and motivating students to plan for their own behavioral changes. We propose establishing evaluation criteria and developing surveys to assess changes in behavior after implementing the intervention program. A report detailing positive behavioral changes and their impact on enhancing well-being can be generated and presented to policymakers in local school boards and advocate groups in non-profit and international organizations.

## 2. Social Interaction and Behavioral and Emotional Issues

The social isolation brought about by the COVID-19 pandemic resulted in a new and stressful daily routine for young people, particularly students who faced significant challenges due to school closures. Schools, which typically serve as the primary venue for social exposure, were no longer available to students. This resulted in limited opportunities for social interactions, particularly those that are crucial for in-person connections [14]. There are generally two groups of students affected: one group generally experienced greater loneliness due to social isolation, and the other group whose social anxiety disorder before COVID was temporarily lessened due to social isolation [14]. Both groups of students met some difficulties when returning to school and restarting their social exposure.

In this section, we conducted a literature search to identify the behavioral issues of social interactions that occurred pre, during, and post the COVID-19 pandemic. Relevant keywords such as “social isolation”, “mental health”, “COVID-19”, “young people”, and “social anxiety” were used to search several databases, including PubMed, PsycINFO, and Google Scholar. In addition, we checked meta-analysis articles on “children, school, and social interactions”. The search resulted in a selective collection of literature that was analyzed and synthesized to provide insights into the impact of the COVID-19 pandemic on social relationships and mental health among children and adolescents. We hope that the literature provides sufficient evidence to support the need for an intervention aimed at cultivating positive behaviors in back-to-school social interactions.

### 2.1. Pre-COVID Social Interactions

Generally, students establish a range of social interactions in schools, including close friendships, cliques and crowd affiliations, and the sense of belonging in larger peer norms [16]. Schools and classrooms are important environments where students fulfill their emotional affiliation needs, which can impact their motivation for cooperation, social responsibility, and increased academic aspiration [17]. Students’ social interactions offer mental support and can provide vital social support for academic success, higher life quality, and development of aspiration. Students’ needs for social interaction and support, particularly from their peers, are core functions schools provide to students and largely contribute to their well-being.

Several meta-analysis studies have evidenced the importance of social interactions with others. For example, Schwartz-Mette et al. [18] examined 16 meta-analyses with 233 studies and found that friendship quality and peer support were closely linked with loneliness and depression. Durlak, Weissberg, and Pachan [19] reported 68 afterschool programs requiring peer support that enhanced children’s and adolescents’ positive social behaviors and reduced problem behaviors. Additionally, students regard positive interactions with teachers as crucial for their well-being and identify social relationships with peers and teachers as a crucial area for improvement in students’ grades [20].

In contrast, recent meta-analyses have provided evidence that social interaction through social media and the internet may have a negative impact on children’s well-being. Prizant-Passal et al. [21] identified 22 studies including more than 13,000 youths and found that social anxiety is positively related to internet use (e.g., feelings of comfort online and problematic use of social media apps). Similarly, Appel et al. [22] reviewed meta-analytic evidence and found intensive use of social media apps is positively associated with loneliness, depression, and narcissism. Hancock et al. [23] conducted a meta-analysis of 226 studies to examine the relationship between social media use and well-being. Their findings indicate that there was no overall association between social media use and well-being; however, there was a positive correlation between social media use and anxiety, depression, and social well-being [23].

### 2.2. During-COVID Social Interactions

After emphasizing the importance of school-based social interactions for students, the remote classroom has hindered the channels students can use to effectively establish and maintain these vital relationships. Long et al. suggested that student populations with a large number of newly established relationships are hard to transfer online and thus we are most likely to find a worsening of relationships during COVID-19 [24]. Because of the objective lack of interactions with others, a range of health issues, including anxiety, panic, insomnia, digestive problems, and post-traumatic stress, are increasing [25].

The importance of physical contact and interactions for human development and well-being is particularly evident during childhood and adolescence. Touches, such as hugs, pats on the back, and high fives, can activate the release of oxytocin, a hormone associated with positive emotions and social bonding [26]. Lack of physical contact with peers may lead to feelings of isolation, loneliness, and disconnection, which can negatively impact mental health and wellbeing [26,27,28,29]. Playing together, practicing social skills, and learning through direct experience are important for the social, emotional, and cognitive development of children. In the absence of physical interaction due to social isolation, developmental delays, impaired social skills, and poor academic performance may occur [12,27]. Research has shown that prolonged physical isolation is associated with an increase in stress hormones, such as cortisol, which adversely affect physical and mental health, causing symptoms such as fatigue, insomnia, and anxiety [30].

Due to COVID-19’s strict social isolation, students’ opportunities to meet friends and peers are involuntarily restricted under the home schooling situation. Students must continue their schoolwork without any physical contact and perceive a lower level of social support from their teachers and peers [31]. Moreover, students are isolated from their social groups, such as organized sports or arts-related extracurricular activities and student clubs and associations [31]. Consequently, more than one-third of adolescents reported a high level of loneliness, accordingly correlated with symptoms of depression and social anxiety disorders [12]. A Canadian study revealed that 86% of parents reported their children experiencing a lack of social connections and identified socializing as their top priority for returning to in-person schooling [32].

Because of the mandatory isolation, young people increased their need to be part of the virtual community and largely expanded their time using social media [33]. Many researchers and organizations suggest that people use social media to keep connecting with social networks and prevent the symptoms of loneliness; however, sometimes excessive social media use can also lead to addiction-like behavior [33]. While virtual social interactions through text and social media have been touted as a way to mitigate the impacts of social isolation, researchers agree that these virtual connections cannot fully replace in-person interactions. In fact, students who engage in greater virtual socializing have reported higher levels of loneliness and depression [34].

### 2.3. Post-COVID Social Interactions

The COVID-19 pandemic has approached its final stage, our daily lives are back to normal, and students are back to school. However, social interactions have not returned to the level those young people expected. The New York Times reported that about 9–10% of young adults and adolescents in the US have a social anxiety disorder that was intensified through months of isolation, and some of them faced social withdrawal and developed reclusive habits [35]. Researchers stated that we might see a higher rate of social anxiety than before COVID since we are starting to socialize more [35]. Although most people are excited about the ‘return to normal’ situation, nearly 49% reported they felt anxious about returning to in-person interaction once the pandemic ended [36]. The core issue is that many people are trying to avoid in-person interactions because they are not used to doing so in the post-COVID era.

Zoom classrooms and social media transformed our way of living and made students not need to interact with people. The first behavioral issue is that students have relied on online interactions more; however, COVID-19 led to more problematic internet use (PIU) and further caused loneliness [37]. Costa et al. [38] stated that PIU leads to higher use of social media and online communications is related to the sense of loneliness because humans tend to recognize satisfying social interactions when feeling rich sensory information and bodily feedback during face-to-face interactions. As homeschooling during the lockdown period prompts people to escape to the online world, it increases social media addiction and the temporary positive emotions produced by social media. Homeschooling makes people want to be permanently immersed in the online world and makes it harder to perceive the emotional value of other methods of social interaction [39].

Although many young people are used to the easy way of online social interactions, research shows in-person interactions are crucial for improving people’s mental and physical well-being and sense of belonging [40]. Adolescence has a particular need for time for social interaction to build relationships outside the family, and the decreased in-person social interaction may impact social skills [41]. The affected social skills can further affect students’ social relationships and well-being. To improve social relationships and reduce loneliness, students need to pay attention to in-person interactions since physically getting together for intimate touch, physical comfort, and reinforcing interactional norms are fundamental needs for people’s well-being [24]. Additionally, engaging in nature can be a meaningful activity that may help alleviate loneliness among individuals [42].

In addition, many students have found the transition from virtual learning to in-person learning challenging, particularly those from minority groups who are experiencing microaggressions and discrimination. In remote learning, minority students may have felt secure and safe in their own homes, shielded from the daily slights and negative experiences they encounter at school. These individuals, however, are once again exposed to an environment where microaggressions are likely to take place, and the emotional toll can be significant. As an example, Asian American students reported higher rates of discrimination and microaggressions related to COVID-19, including being coughed at intentionally, racial insults, and physical intimidation [43]. A study conducted by Kim et al. [44] found that Asian American students encountered similar microaggressions during their internships outside of school. The experience of microaggressions may result in increased stress, anxiety, and a reduced sense of belonging, which can negatively impact academic performance [45,46].

## 3. Intervention

Since COVID-19 transformed how students establish and maintain their social relationships, thereby increasing loneliness by spending more time on social media and online communications, schools and education agencies should provide support to help students develop social skills and social relationships in the post-COVID era. Accordingly, a school-based intervention should be created to support students in overcoming the hard back-to-school transition. The intervention is a four-section workshop based on the participation principle to encourage students to spend less time on online social networking and instead use more time on in-person social interactions. The intervention workshop included (1) self-reflections and discussions, (2) motivational interviews, (3) informative treatment, and (4) workbooks. The program starts with a group component involving self-reflection and group discussions among participants. Afterwards, each participant receives peer motivational interviewing and an individual workbook for outlining their behavioral plan. School teachers can lead the intervention workshop with a total length of about 90 min. Teachers are expected to participate in the administration of the program. The program was designed for school-age children and adolescents.

The program was developed by the authors of the article who were trained in public health, psychology, and education. The four components were carefully chosen based on both theoretical and practical considerations. For instance, self-reflection has been proven to be a valuable intervention method for promoting self-awareness and exploring social interaction among youth with mental health issues [47]. On the other hand, motivational interviewing and workbook interventions have demonstrated effectiveness in areas such as career decision making for students [48], school achievement [49], and overall well-being of children [50]. In the next section, we provide comprehensive information on the theoretical, methodological, and practical aspects of program design. It is important to note that these frameworks and interventions are not exhaustive, and other strategies can be considered based on the needs of the targeted age groups (e.g., school-aged children and adolescents) and the professionals involved in the intervention. Ultimately, the success of the intervention will depend on the implementation of evidence-based strategies and a multi-disciplinary approach that includes collaboration between mental health professionals, teachers, and families.

### 3.1. Theoretical Orientation of the Intervention Design

A school-based mental health program is an essential component of mental health interventions, given that students spend most of their time at school and that schools play an important role in developing students’ social and cognitive skills [51]. A number of studies have shown that schools can establish positive and effective programs to address the mental health needs of students. Australia’s MindMatters program provided schools and teachers with the necessary skills for promoting student well-being at the national level [52]. In addition, a variety of school-based interventions have been successfully implemented to address the mental and behavioral health of students. The ‘I can problem solve’ program, which is a series of lessons designed to improve problem-solving skills in primary school students, has shown evidence of effectiveness [53]. An educational program called Zippers Friends, which develops students’ coping skills, has been found to significantly reduce mental health issues among students [54]. Moreover, ‘The Good Behavioral Game’ has been shown to be an effective school-based program for reducing substance abuse and antisocial behavior [55].

Furthermore, the emergence of positive education (e.g., positive psychology, growth mindset, and grit) presents an opportunity for schools to play a more active role in educating students’ academic excellence as well as their well-being in parallel [56,57,58,59,60]. Positive education that incorporates well-being education into the classroom and day-to-day school life reduces students’ mental health disorders, depression, and anxiety, and improves their interpersonal relationships and school engagement [56,57]. A growth mindset can also promote greater learning and resilience, particularly in educational settings [58]. Those students with a growth mindset are more likely to embrace challenges, persist despite obstacles, and achieve greater success than those without [58]. Additionally, grit, which is defined as a combination of passion and perseverance toward long-term goals, has been shown to be associated with the outcomes of childhood education [59]. Students who possess more grit perform better academically and are more likely to graduate from high school and college [59]. Furthermore, Positive Behavioral Interventions and Support (PBIS) provides a framework for designing and implementing school-based interventions for students to improve their mental and behavioral health [60].

An educational intervention is designed to facilitate students’ intention to change their focus to in-person social interactions. First, students should accept the anxiety and face the fear of re-entering social situations. Second, students should develop motivation for establishing and developing their social relationships in an in-person mode. Third, students should understand how to fit in and what they can do in social situations. Fourth, students should commit to encouraging themselves to better facilitate in-person social interactions.

The intervention design is based on the participation principle, aimed at promoting public engagement by enhancing participants’ resolve to achieve their objectives [61]. The objective is to empower participants to fully comprehend the challenges, confront their fears, acquire the necessary skills, and be prepared for change. According to Backer [62], eight principles for behavior change through increased participation exist, including the formation of a strong positive intention or commitment to act, the perception that the benefits of change outweigh the drawbacks, the acquisition of the necessary skills and belief in one’s ability to perform, and the minimization of environmental obstacles to change [62].

Meanwhile, to help students make positive changes, the present intervention design is adapted to the model of motivational interviewing, including engaging, focusing, evoking, and planning. These steps are critical in facilitating behavioral change and promoting active participation, as described by Miller and Rollnick [63] in their model of motivational interviewing. Engagement is crucial in establishing participants’ perceptions of the situation, while focusing helps participants gain clarity on the desired change. Evoking elicits participants’ motivations for change and planning assists in creating specific plans for behavior modification. Therefore, serving different aims of participants’ beliefs and capabilities, the intervention designed four sections for cultivating a comprehensive behavioral change, integrating individuals’ behavioral changes as well as teachers’ involvement in the school context.

### 3.2. Self-Reflection and Discussion

The first section of the intervention is self-reflection. The group-based component involves the formation of discussion groups consisting of individuals who engage in a 30-min self-reflection and group discussion. The main aim is to raise students’ awareness of the situation and dilemma and should focus on the engagement principle. Teachers can help students recognize what happens, what their needs are, and what is going wrong. Students are expected to understand and face their anxiety about social interactions and become aware that anxious situations normally happen. This will help them actively deal with their social anxiety.

Discussion questions are developed based on the concept and techniques of Self-Reflection Resilience Training (SRT), which encourages participants to self-reflect on stressor events. The intervention focuses on promoting resilience, as the ability to swiftly bounce back from stressors has been demonstrated to have a positive impact on reducing anxiety levels in participants [64]. SRT encourages self-focused attention and simultaneously reduces harmful brooding and focuses on positive self-development [64].

Starting with engaging participants, teachers can facilitate this section by providing students with three questions and 10 min to consider their current social situation and dilemmas.

1. Have you been struggling with social interactions online or in-person on any level during COVID or after returning to school? How do you think COVID has influenced your social relationships?
2. Have you faced social anxiety or social awkwardness of any level since returning to school? What are your emotional, physical, behavioral, and cognitive responses to social situations?
3. Are you dissatisfied with your current social interactions and social relationships of any level? What is your goal for developing satisfying social relationships?

After 10 min of preparing the questions, students can have 20 min to share their thoughts with other group members. During the process, students can easily feel peer support, become aware that their anxiety is normal, reduce their negative feelings and establish a sense of belonging to the group. Then, students can recognize their actual needs in social interactions and focus on developing better social relationships.

### 3.3. Motivational Interview

The second section of the intervention is the motivational interview. This section happens on a one-on-one peer support basis, and students can have 30-min in this section. A student can spend 15 min asking questions of the other student, and vice versa. This section is aimed at letting students recognize how they can improve their relationships and what their motivations for the change are, based on the focusing on evoking principle. Teachers should try to ensure all students know what to do and why they should do it for positive interactions. The motivational interview is expected to help students improve their social skills in the post-COVID era.

Although the motivational interview is usually conducted by professionals, this workshop adapts its concepts and techniques in a peer support setting [48]. The questions direct students to acknowledge their recent behavioral changes due to COVID-19, their declining social abilities, and their tendency to avoid social interactions. Then, students are guided to consider how they spend time on both online and in-person social interactions and how they feel loneliness during their social interactions. It is anticipated that the intervention will enhance students’ resilience to stress and foster a positive school environment, ultimately leading to improved well-being and academic performance.

After engaging students by making them aware of the general social interaction dilemma they face, the one-on-one peer interview can let students focus on their individual problems and evoke their motivation for changes. Teachers can provide a question list for students to communicate.

1. How have your social skills and behaviors in social interactions changed since COVID? How have you changed your time consumption on social interactions online and in-person?
2. How do you feel about your online and in-person social interactions, respectively, since COVID? Such as, fulfilling, exciting, lonely, or anxious?
3. What do you want to obtain from social interactions, both online and in-person, respectively?
4. Do you feel any of your needs or wants are not fulfilled? What are your constraints for establishing or maintaining social relationships to fulfill your needs?
5. Have you experienced exciting and memorable in-person social interactions? Why do you think these interactions matter?
6. If you are going to make a strategic plan for improving your social relationships, how will you better spend your time with your social networks, online and in-person, respectively? How do you plan to interact with your social networks?

Each student can have 15 min to respond to six questions about their behaviors, needs, aims, and plans for online and in-person social interactions. Students are expected to recognize their ideal social relationships and ways of social interactions. Next, students are invited to highlight their wants and make plans for the behavioral changes of meaningful social interactions, especially how to spend more time on and better utilize in-person interactions. For example, some minimal trials can be attending a student club, going to a home party, or having dinner with friends.

According to a meta-analysis conducted by Rubak and colleagues [65], interventions using a brief MI of only 15 min have been found to be effective in promoting various behavioral changes. Specifically, the meta-analysis found that 64% of interventions using a brief MI were effective in achieving desired behavioral outcomes. This suggests that even a short MI can have a positive impact on behavior change, making it a potentially useful tool for professionals in a variety of settings.

The approach of motivational interviewing (MI) has been traditionally used by psychologists and professional counselors to facilitate positive behavior change. However, recent research has suggested the potential for non-professionals, such as teachers and peers, to be trained in MI techniques and conduct effective interventions. Gai et al. [48] conducted a study in which peer coaching was used as a vehicle for MI and showed the effectiveness in promoting students’ career adaptability. Similarly, Svensson et al. [66] demonstrated the effectiveness of MI administered by teachers to address challenging behaviors and enhance students’ motivations. In both studies, the use of MI by non-professionals was found to be effective, indicating the potential for expanding the reach of MI interventions beyond traditional therapeutic settings. It is important to note, however, that the effectiveness of MI may vary depending on the specific context and individual characteristics, and other factors may need to be considered when selecting and implementing interventions.

### 3.4. Workbook

**Figure 1:**
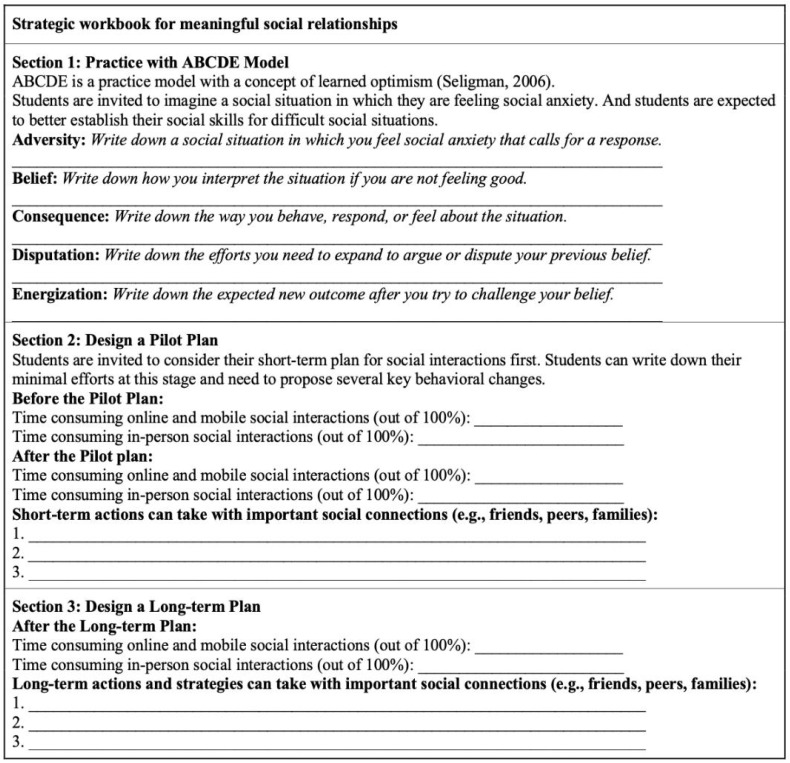
Strategic Workbook Form [56].

The third section is to ask students to develop an individual workbook to detail the behavioral plan for improving social relationships by spending more time on in-person interactions. Focusing on the planning principle, teachers can provide the following template for students to complete their individual plans focusing on when and how to change instead of whether and why [63]. Students can spend the final 30 min developing commitments to making changes and designing a specific plan of action (See Figure 1).

After completing the workbook, students are expected to establish their own strategy and motivation to spend more time on in-person social interactions and do something meaningful and exciting with their social connections. Changing students’ behavior from excessive social media use to proactive in-person social interactions can effectively help students cope with the post-COVID social skills absence and social anxiety.

### 3.5. Organization Readiness and Informative Treatment

After completing the workshop, students will have a comprehensive view of their post-COVID social dilemmas to be better motivated to act and improve their social relationships, especially in the back-to-school context. However, besides the individual work students should do to proactively interact with others in-person, the school should also play a vital role in establishing a warm and supportive environment for facilitating the changes on the organizational level [67]. First, teachers can serve as leaders in establishing the dynamics of the classroom within the school context. Second, an environment that facilitates change with minimal impediments is crucial for individual behavioral changes to occur.

Teachers can motivate students by using several strategies recommended according to the meta-analysis on the influence of affective teacher–student relationships [68]. For example, teachers can promote in-person social interactions by giving a mini-lecture or guidance in the classroom [68]. They can express the school’s encouragement for students to enhance their social life and make it an exciting and fulfilling experience [68]. To achieve this, teachers can make learning activities more interactive and foster collaboration among students. Additionally, teachers can provide extracurricular opportunities for students to participate in clubs, sports, and student associations. They should also emphasize the school’s concern for students’ mental well-being and encourage students to seek help when facing challenges.

### 3.6. Evaluation

The proposed intervention aims to increase students’ time consumption on in-person social interactions. By implementing the intervention workshop in the classroom, students are expected to spend more time engaging in in-person interactions and to improve their social relationships.

Intervention workshops can be implemented in different schools. We recommend randomly selecting schools for an intervention or a control group as the first step in ensuring that the intervention results are effective and generalizable. As part of the selection process, factors such as school size, geographical location, and student demographics should be considered in order to ensure that the intervention is representative of the larger population of schools in the area. Although using a school outside of the area as a control group is possible, it may not be the best option in all cases. In choosing a control group that is located outside of the intervention area, other variables that are unique to that area may not be taken into account. Instead, a similar school within the same area that does not receive the intervention may be a more effective alternative. In this way, the control group will have similar contextual factors, such as community resources and social support. If researchers plan to use a particular school as the site of the intervention, students should be randomly assigned to the intervention and control groups. The random assignment of students can be accomplished using random blocks or other appropriate methods in order to ensure that the intervention’s effectiveness can be accurately measured. Six months after the intervention, an evaluation can be conducted to compare the reports from the intervention and control groups. The following survey questions were developed to measure and evaluate students’ behavioral changes in social interactions. Students can be invited to complete the survey 3 months and 6 months after attending the intervention workshop. To examine the impact of the intervention on the outcome variables, repeated measures ANOVA can be conducted. Time (pre-post) and group interaction effects (intervention and control) on outcome variables should be examined to demonstrate the effectiveness of the intervention.

Some Possible Measures:

1. How many hours a day do you approximately use any form of social media? (e.g., Instagram, Facebook, Snapchat, Twitter, etc.)? [69]
2. How many times do you approximately visit any social media sites or apps, using any device per day? The options are ‘More than 10 times per day’, ‘2 to 10 times per day’, ‘Once per day’, or ‘Less than once per day’ [69].
3. The 14 items Warwick Edinburgh Mental Well-being Scale [70].
4. The four items Subjective Happiness Scale [71].

All measures have been used and validated in samples of children and adolescents across multiple countries.

### 3.7. Limitations

A potential limitation of the proposed interventions is the lack of teacher support. Teacher involvement is often necessary in behavioral interventions, including planning and implementing the intervention, monitoring student progress, and providing ongoing support to students. It is possible, however, that some teachers lack the necessary training, resources, or time to effectively implement the intervention. Furthermore, teachers may not be able to fully engage in the intervention because of competing priorities, or may not see the value of the intervention. The intervention may not achieve its intended outcomes if the teacher does not receive adequate support for it.

Another limitation of the intervention is the heterogeneity of the student population at a given school. It may be necessary to adapt the intervention in order to meet the needs of all students, taking into account their individual differences. Some students may require specialized care because of underlying mental health issues, while others may need basic strategies to improve their social interactions. Additionally, students may come from diverse cultural and socioeconomic backgrounds, which may impact their response to the intervention. Thus, it may be necessary to design and implement interventions in a tailored manner to ensure that the program is beneficial to all students.

## 4. Conclusions

This intervention program is expected to result in a significant improvement in students’ social skills, face-to-face social interactions, and, consequently, a boost in their social relationships and overall well-being. Since mental health services are in a significant shortage in the post-COVID era [7,8], effective school-based interventions can largely reduce the burden of mental health services and significantly reduce students’ cost of receiving mental health services. Schools are encouraged to implement intervention workshops and tailor them to their specific needs. Moreover, schools can prepare an organizational strategy to better motivate students to engage in school-based social interactions. For example, an update in the curriculum, a training session for school staff, and a guidebook for parents can be great ways to establish a better school environment for students to engage with each other.

Meanwhile, this perspective article is to call attention from the policymakers of schools to implement interventions for coping with students’ social anxiety and improve students’ social skills in the post-COVID era. It is expected that the newest education policy can focus on students’ well-being and the intersection of education and health policy. Global policymakers from both education and public health sectors can collaborate to design school-based interventions to enhance students’ physical, mental, and emotional well-being, mitigate the negative effects of school closures and social isolation, and broaden the role of schools to cater to students’ comprehensive needs and support their growth in the post-COVID era.

## Data Availability

Not applicable.
